# Recurrence of Laryngeal Hemangioma in an Adult: A Case Report

**DOI:** 10.7759/cureus.31090

**Published:** 2022-11-04

**Authors:** Twisha S Shukla

**Affiliations:** 1 Paediatrics, California Institute of Behavioral Neurosciences & Psychology, Fairfield, CAN

**Keywords:** case report, recurrent, benign vascular tumor, vocal cord lesion, hoarsness, airway anomalies, laryngeal lesion, infantile hemangioma, laryngeal hemangioma

## Abstract

Laryngeal hemangiomas in adults are uncommon. Laryngeal hemangiomas are more common in children, and if treatment is required, it is treated with propranolol. An eighteen-year-old female with rare glottic hemangioma extending to a supraglottic region presented with shortness of breath and hoarseness of voice. Although complete resection of the hemangioma was impossible due to its proximity to the vocal cord, it was treated with Microlaryngoscopy ablation with a CO2 laser. She has been followed up for over 10 years. The patient has had three recurrences, which have also been described. The cause of recurrence after treatment and the trigger for the increase in the size of the hemangioma is yet to be identified. The patient is now stable after her last Microlaryngoscopy and CO2 ablation. In conclusion, a near complete resection of hemangioma is required to prevent regrowing of hemangioma.

## Introduction

Hemangioma is a benign tumor made up of blood vessels. The Greek suffix “oma” means a cellular proliferation of a tumor, and “hemangioma” is related to blood vessels. So, hemangioma refers to a tumor of the blood vessel. They are more commonly seen in pediatric groups and are usually benign. They first undergo a proliferative phase in which hemangiomas undergo rapid proliferation during the first few weeks/months of life, and they last for 12-18 months. After that, in the involution phase, the size decreases, and complete resolution can take up to 12 years. In the adult, they can occur at any age and do not follow the proliferation and involution phases. In Infants, laryngeal hemangiomas are almost exclusively capillary type. They commonly involve the subglottis, the narrowest portion of the pediatric airway [1], whereas, in adults, they are most commonly found in supraglottis. As most laryngeal hemangioma in children regresses over time, they do not need treatment. In adults, treatment is usually required. This article discusses the rare case of recurrent laryngeal hemangioma in an adult female and its treatment.

## Case presentation

An otherwise healthy 18-year-old girl started complaining of difficulty breathing after exercise and a change in her voice for three months. Difficulty breathing has worsened over the past month, even with mild to moderate walking. She had no history of smoking, infection, past intubation, trauma, or voice overuse. Her physical examination, blood work, and chest X-ray came back negative. X-ray of the soft tissue neck shows a well-defined space-occupying lesion the in the vocal cord region. A polypoidal lesion was visualized with direct laryngoscopy. On Multi-slice Computed Tomography (MSCT) scan, a well-defined soft tissue polypoidal lesion from the true vocal cord involving posterior commissure on the left side and projecting into rima-glottidis was identified, narrowing the airway. The size of the lesion was 12.5*9*10 mm. Tracheostomy and micro laryngeal surgery with co-ablation were done under general anesthesia. A pathology report identified capillary-type vascular proliferation - hemangioma- in supraglottis. On postoperative day 5, her tracheostomy tube was removed, and she was discharged. But the following day, the patient was re-admitted with complaints of breathlessness and was taken for direct laryngoscopy with the removal of slough and re-insertion of the tracheostomy tube. The histopathology report showed a post-surgical reaction. A follow-up MRI two months later showed the lesion size to be 10*6*7 mm.

**Figure 1:**
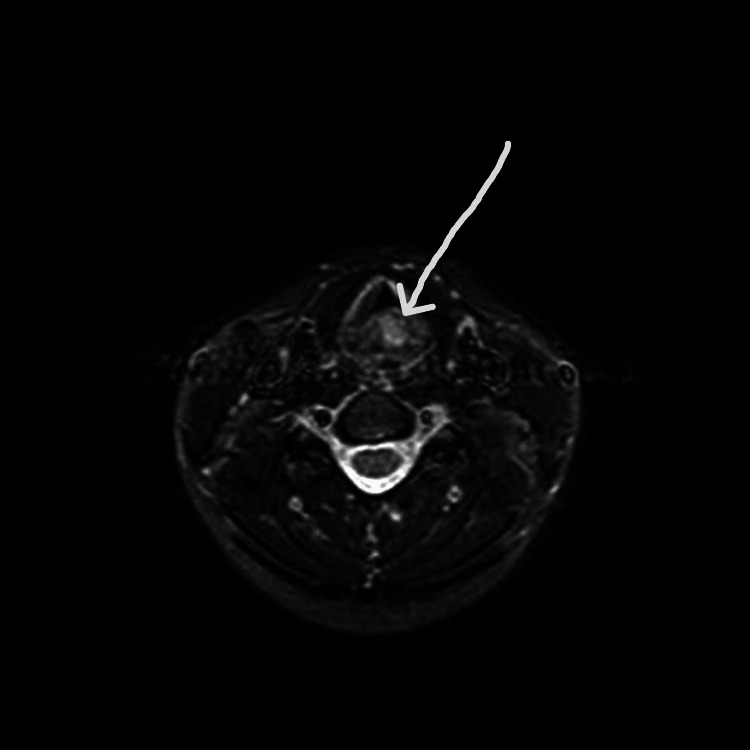
MRI showing hemangioma during the first presentation. The arrowhead is pointing towards the tumor.

After five years, patients started developing difficulty breathing during exertion. MRI showed an increase in the size of the hemangioma to 10*13*13 mm at the level of posterior commissure in midline projecting in the laryngeal ventricle and supraglottic larynx. Her symptoms kept worsening, and a repeat MRI showed an increase in the lesion size to 11*14*15 mm (fig 1). Micro-laryngoscopy for biopsy and debulking using a CO_2_ laser was done. Biopsy results showed lymphoplasmacytic cell infiltrate with a thin-walled capillary. After three months, patients started feeling difficulty in breathing. MRI with angiography showed an increase in supraglottic laryngeal hemangioma to 13*14*16 mm. It showed a well-defined homogenously enhancing lobulated submucosal lesion at the level of posterior commissure in midline projecting in the laryngeal ventricle and supraglottic larynx and abutting inferior aspect of the left aryepiglottic fold as shown in Video 1, consistent with a hemangioma. Angiography showed a small arterial channel at a posteroinferior aspect of the lesion, possibly arising from a branch of the superior thyroid artery. A tracheostomy with debulking of the laryngeal mass was done. Video 2 Shows endoscopy following surgery. A Follow-up MRI three months later showed a residual lesion of size 4.9*4.4*6 mm.

**Video 1:** Pre-Operative Endoscopy showing Hemangioma

**Video 2:** Post-Operative Endoscopy

After three months, the patient started having symptoms. She had difficulty breathing with a tracheostomy tube in situ. MRI showed a residual homogenous signal intensity lobulated submucosal lesion at the level of posterior commissure in midline projecting in the laryngeal ventricle and supraglottic larynx and abutting inferior aspect of left aryepiglottic fold. A video laryngoscopy showed that the mass in the inter arytenoid region was extending into the subglottis. Near total removal of mass with micro laryngoscopy with CO_2_ laser ablation was done. The biopsy report showed granulation tissue with spindle cell proliferation. Her last endoscopy is shown below in figure 3 on the right side. The patient is now symptom-free. The patient is advised to follow up every five years and immediately if any symptoms develop.

## Discussion

Hemangiomas are benign vascular tumors and common infancy tumors [1]. Hemangiomas can be classified according to histology, anatomical location, or the age of occurrence. Hemangiomas that grow by endothelial cell hyperplasia should be differentiated from localized defects of vascular morphogenesis called vascular malformation. The International Society for the study of vascular anomalies (ISSVA) classified vascular tumors from vascular malformations based on their clinical appearance, radiological features, pathological features, and biological behavior [2]. Vascular neoplasms have increased endothelial turnover, whereas vascular malformations are structural abnormalities of the capillary, venous, lymphatic, and arterial systems that grow in proportion to the child. According to this classification, magnetic resonance angiography of hemangioma shows a well-delineated tumor with flow voids, whereas vascular malformation shows hyper-signal on T2 sequences and may show a heterogenous signal on T1-weighted MRI [3]. Moreover, hemangioma seems homogeneous hyperintense on T2-weighted MRI, which is usually isointense compared to T1-weighted MRI [3].

In general terms, hemangiomas are benign true vascular tumors characterized by increased vascularization, representing 7% of all benign tumors in infancy and childhood [4]. Hemangiomas of the larynx can be divided into infantile and adult. Laryngeal hemangioma presents as abnormal cry, persistent stridor, and chronic cough and is accompanied by a cutaneous lesion [5]. The differential diagnosis of airway obstruction in the pediatric population is laryngomalacia, vocal cord paralysis, subglottic stenosis, laryngeal webbing, laryngeal atresia, laryngeal cleft, saccular laryngeal cyst and laryngocele [5].

As infantile hemangiomas are known to undergo rapid proliferation followed by slow involution, the primary goals of treatment should be to provide an adequate airway during the proliferation period, avoid life-threatening obstruction and minimize therapies that may result in long-term complications. The medical management of infantile hemangioma includes systemic corticosteroids, which can halt the progression of hemangioma during the proliferative phase. However, it comes with adverse effects like cushingoid appearance and hypertension. Interferon treatment is reserved for a refractory type of infantile hemangioma. Vincristine has also been used successfully, but only limited data is available. Surgical management includes laser ablation and tracheotomy [6]. Propranolol has been reported to decrease the cutaneous and airway hemangioma size drastically. As the recommended dose of propranolol is lower than that used in cardiovascular disease, there are only a few adverse effects [7]. But one has to watch for bradycardia, hypotension, hypoglycemia, and worsening of reactive airway diseases [6].

Adult hemangiomas are rare and, more often, cavernous forms [8,9]. The patient described here had a capillary type of hemangioma, and capillary hemangioma has well-circumscribed and smaller in size than a cavernous hemangioma.

In an adult, a laryngeal hemangioma can occur at any age. The etiological factors include cigarette smoking, vocal abuse, and laryngeal trauma. There are few to no symptoms associated with it. Although rare, it is usually found at or above the level of the vocal cords. Irritation with the region is the only main symptom. It can present as hoarseness, hemoptysis, shortness of breath, dysphagia, aspiration with thin fluids [10], globus, acute respiratory distress [11], sudden collapse, and death [12]. There is no skin lesion present with an adult type of hemangioma. Adult laryngeal hemangioma involves the supraglottic region. The presentation usually includes dysphagia, dysphonia, shortness of breath, and recurrent bleeding [13]. Treatment options depend on the age of the patient, anatomical location, size, and type of lesion. For infants with hemangioma causing no symptoms, spontaneous regression can be expected. Adult hemangiomas usually are not progressive, so clinical observation is the best management strategy [8]. For large or symptomatic hemangioma, systemic steroids, corticosteroids injections, ethanol injections, cryosurgery, radium or gold implants, interferon treatment, laser surgery, and ultrasonic scalpel [14] can be used [8,14-16].

In one paper, hemangioma sized 3*2.2*2.5 cm was excised with CO2 laser in continuous mode and injected Depo-Medrol (corticosteroids) to shrink the remnant of the hemangioma [10]. Although in this case, the hypopharyngeal part of the mass was left untreated because of surgical hazards to injure large blood vessels. Although, there was no recurrence or any complication noticed in the 2-year follow-up period [10].

Argon plasma coagulation can be used, which has high safety profile due to reduced depth of penetration [13]. A paper published on ultrasonic scalpel dissection reported being a safe, effective, and less invasive method for treating large laryngeal hemangiomas [14].

## Conclusions

In conclusion, adult laryngeal hemangiomas are very rare. An adult supraglottic hemangioma can be treated in one or more stages with CO_2_ laser excision and an ultrasonic scalpel. The causes and triggers for recurrence are still unknown. This case report suggests that complete or near complete resection is necessary to avoid regrowth. However, few articles have suggested use of intralesionalnal corticosteroids after excision may prevent a recurrence. More studies need to establish the guidelines for treating laryngeal hemangiomas in adults and how we can prevent their recurrence.
